# The talk debrief experience: Intervention in prehospital personnel care during the Covid-19 pandemic

**DOI:** 10.3934/publichealth.2024041

**Published:** 2024-06-24

**Authors:** Olga Malas, Xavier Perez-Cuit, Jordi García-Sicard, Andrés Cuartero, Gemma Cuartero

**Affiliations:** 1 Department of Psychology, Sociology and Social Work, University of Lleida. Avinguda de l'Estudi General, 4, 25001 Lleida, Spain; 2 Emergency Medical System, Department of Health and Social Security, Health Department Government of Catalonia. C. Pablo Iglesias, 101-115, 08908 Hospitalet de Llobregat, Spain

**Keywords:** talk debrief, debriefing, prehospital personnel, distress, anxiety, mental health

## Abstract

This study focused on the TALK Debrief Experience in the prehospital personnel (*n* = 1521) of a western Spanish healthcare region during the COVID-19 Pandemic. The study aimed to apply the TALK clinical debriefing intervention to out-of-hospital clinical staff during pandemics; identify their emotions, thoughts, coping strategies, and solution proposals; determine their demands for improving well-being and coping ability; and disseminate valuable knowledge for addressing trauma in similar situations. The study employed a qualitative methodology within a participatory action research (PAR) framework, conducting group discussions (*n* = 375) and employing the TALK clinical debriefing method as the guiding framework for the sessions. The discussion group meetings were facilitated by psychologists (*n* = 67) who had received training in this intervention technique. Various emotions were identified during the sessions, including fear of contagion, lack of control and security, work-related stress, and ethical dilemmas. Proposed solutions and coping strategies addressed increased security measures, promotion of social distancing, stress and anxiety management, and clarity in procedures and provision of protective equipment. The study also highlighted additional demands such as the need for clear information, psychological support, and changes in work practices like reducing strenuous shifts. In conclusion, despite study limitations, such as the lack of long-term follow-up, it emphasized the importance of comprehensively addressing well-being and working conditions during health crises.

## 1. Introduction

The coronavirus disease, known as COVID-19 or SARS-CoV-2, reached Spain in February 2020 and escalated progressively. From the second week of March onwards, the growth became uncontrollable in some of its regions. For instance, in the Autonomous Community of Catalonia, located in the northwest of the country, on March 11, 2020, the Health Minister reported an exceptional outbreak in the city of Igualada. A significant percentage of those infected were healthcare workers. The number of infections in the region progressively increased in the following days, making it one of the most significant epicenters of the outbreak in the country.

Repeated exposure to distressing and potentially traumatic events has a significant impact on the physical and mental health of healthcare professionals [1]. Considering the situation in the area and the high percentage of healthcare personnel infected, the need arose to activate a group of psychologists from the Medical Emergency System to provide support to prehospital professionals who were on the front line, crucial in the healthcare system during crises. The aim was to reduce anxiety and the risk to mental health among healthcare staff through psychological interventions aimed at alleviating distress from their continuous exposure to contagion. This involved emotional support, identifying the needs and concerns of healthcare personnel, and proposing specific measures to improve their well-being and coping capacity. Additionally, it aimed to disseminate useful knowledge and practices to other professional collectives and organizations that may face similar stress and trauma situations. After several expert meetings, it was decided to use the TALK clinical debriefing as the basis for achieving this goal.

Conceptually, clinical debriefing is an interprofessional meeting that occurs after a critical event, providing nurses and other healthcare team members with the opportunity to review and process their experiences [2],[3]. The Critical Incident Stress Debriefing (CISD) report was developed in 1983 to address stress management in high-risk occupations such as police, firefighters, and emergency responders [4], and tends to be confused with the clinical debrief [5]. CISD and other similar interventions promote emotional processing through detailed recounting of the traumatic experience to normalize the response to trauma and prepare for possible future emotional and behavioral experiences [6]. This intervention is facilitated as a semi-structured group meeting, a single session, between 24 and 72 hours after trauma exposure, led by a mental health professional and a peer support representative [4]. Despite its widespread use, there has been much controversy surrounding its appropriate use and effectiveness in preventing post-traumatic stress disorder and other psychological consequences [7]. In fact, two longitudinal studies included [8],[9] reported significantly higher rates of PTSD among participants who attended debriefing compared to those who received no intervention. However, in a more recent systematic review, the authors concluded that satisfactory outcomes are achieved when interventions are tailored to meet the needs of their recipients in a format of clinical debrief [10]. Moreover, in clinical settings, debriefing sessions are conducted in groups, transitioning into an operational meeting during which decisions made are reviewed, learning points are identified to enhance the quality of care, and individuals are given the opportunity to offer personal reflections to create a shared narrative of the event they experienced [1] and to support each other [11]. In other words, debriefing is not used as a treatment for post-traumatic stress disorder or as a preventive intervention, but rather as an early opportunity for peer support, normalization of emotional experiences [1], and enhancement of resilience during pandemic challenges and subsequent recovery periods [12]. Team healthcare meetings are another form of gathering that can be misconstrued as a clinical report [5]. Clinical reports differ from meetings in that they are intermittent, sporadic, and unplanned gatherings that occur after critical events, with complex and multifactorial causes, capable of putting patients and/or others at risk of harm [5],[13]. They depend on the situation and are based on needs [5]. Another particularity of these team healthcare meetings is that their main aim has been to enhance clinical outcomes by learning through discussion and reflection on events, and subsequently applying that learning to clinical practice; placing less emphasis on the emotional experiences and well-being of healthcare workers [14].

Clinical debriefing sessions have been associated with numerous personal and professional benefits for nurses and other healthcare team members [3],[15]. Studies on the topic suggest that discussing and interpreting events in the workplace can emotionally benefit healthcare workers, helping to enhance and maintain a resilient workforce during crises such as the Covid-19 pandemic [16]. So, despite the debate surrounding it, the report can be a valuable tool as an affordable, adaptable, and cost-effective intervention for a community of professionals who require emotional support to carry out essential and challenging work [17].

To ensure the effectiveness of the intervention, the clinical report should be interprofessional, and all personnel involved or affected by the critical event (nurses, doctors, healthcare assistants, and others) should attend [2],[18]. The goal is for all team members to process and review the circumstances and impact of a specific event [19]–[21]. In any case, prior studies on emergency personnel recommend debriefing in early interventions to prevent trauma [10], yielding better outcomes when conducted by trained professionals [22],[23] and participation is voluntary [23].

However, significant inconsistencies in the delivery and structure of clinical reports have been described [5]. Hence, the expert group selected the TALK clinical debriefing as a guide for its availability and ease of application. Since the TALK format is based on needs analysis and the subsequent search for practical solutions to these needs, gathering information during the sessions provided a comprehensive framework of the reality of healthcare professionals, allowing access to their thoughts, visions, opinions, and perspectives in a complete and ecological manner. In this context, the specific objectives of this study were:

1. Apply the TALK clinical debriefing intervention to out-of-hospital clinical staff in crisis situations such as pandemics.
2. Identify the emotions, thoughts, practical solutions, and coping strategies of pre-hospital professionals who work on the front lines during the crises.
3. Identify the demands of these professionals aimed at improving well-being and their coping ability.
4. Disseminate useful knowledge and practices to address similar situations of stress and trauma.

The central research question was: What are the effects of repeated exposure to distressing and potentially traumatic events, such as pandemics, on the health of frontline out-of-hospital healthcare professionals, and how useful is the TALK clinical debriefing in addressing their needs, concerns, and coping strategies, and improving their well-being?

## 2. Materials and methods

### 2.1. Participants

The sample consisted of a total of 1521 workers from the Emergency Medical Service of the Autonomous Community of Catalonia (Spain), representing 21% of the total workforce. For sampling, the non-probability convenience sampling method was applied. Of these, 86% were male, 14% were female, aged 18 to 65 years. The participants included physicians (12.4%), nurses (19.1%), ambulance technicians (68%), and helicopter staff (0.5%). A total of 375 discussion groups were formed (see Table 1), with an average of 4 participants per group.

**Table 1.** Number of discussion groups by health region.

**Health region**	**Number of discussion groups**	**Total participants**
High pyrenees	18	117
Barcelona city	52	279
Catalunya central	106	252
Girona	44	184
Lleida	30	101
Tarragona	36	106
Central services	9	55
Northern metropolitan	42	233
Southern metropolitan	38	194
**Total**	**375**	**1521**

### 2.2. Instruments

This tool guides individuals to maintain effective communication among healthcare professionals through centered and constructive conversations with practical outcomes. It encourages sharing reflections meaningfully, inclusively, constructively, and without bias, highlighting positive strategies and behaviors while avoiding negative comments. It focuses on finding solutions, promotes professional communication, respects and values the contributions and perspectives of all involved, and takes a step-by-step approach, starting with small things and achievable goals [11].

### 2.3. Procedure

This study was conducted using a qualitative methodology in a group discussion format, aligning with a participatory action research (PAR) approach. The TALK clinical debriefing guide served as a script for the sessions. A team of 67 specialist psychologists, who received specific training in TALK clinical debriefing beforehand, was deployed to conduct the intervention. The researchers played an active role in carrying out the study, facilitating group sessions, providing guidance to participants, documenting the results, and analyzing the collected data, thus ensuring the integrity and effectiveness of the research process. They traveled to different bases of the Emergency Medical Services in the Autonomous Community of Catalonia (Spain), accompanied by a local leader. The discussion group meetings lasted 30–45 minutes each, during which the TALK debriefing tool was introduced, explained, and applied. The outcomes of the sessions were documented in reports. A structured approach was employed for report presentation, using a standardized form completed by the team of psychologists during and after each meeting. The rationale for using this method of data collection lies in its structured approach, which ensures consistency and comprehensiveness in capturing the outcomes of the group discussions. By employing standardized forms completed by trained psychologists, we can systematically record the participants' insights, emotions, and proposed solutions, facilitating thorough documentation and subsequent analysis. This method enhances the reliability and accuracy of data collection, enabling us to derive meaningful insights from the sessions.

### 2.4. Ethical considerations

This study was reviewed and approved by the Ethics Committee of the Institut d'Investigació Sanitària Pere Virgili (IISPV) and the was the responsibility of the Emergency Medical Services of Catalonia (Spain). The study followed the principles outlined in the Declaration of Helsinki. The specialist psychologists informed the participants about the study objectives prior to implementing the intervention. Participation was voluntary, and individuals had the freedom to withdraw from the session at any point. Oral informed consent was obtained from all participants before they joined the discussion groups. The responsible psychologist documented this process in the relevant report, which was signed by all attendees.

### 2.5. Analysis data

The analysis of results followed the qualitative analysis phases outlined by Bardin [24]: pre-analysis, category system formation, coding, and analysis. In the Pre-analysis phase, the reports were reviewed to gain a general understanding of the content and characteristics of the data. In the subsequent Category System Information phase, key themes, patterns, and concepts were identified, and a system of categories or themes was developed to organize the information. This allowed for the Coding phase, where data was labeled based on specific concepts relevant to the research objectives. To facilitate this task, reports generated during the sessions were loaded into the QSR NVivo 12 Plus software for coding and analysis. Using content analysis with constant comparative coding, a codebook was developed from participant-identified priorities. During the coding process, researchers collaborated to analyze content and ensure consistent code applications. Disagreements were resolved through consensus, and code definitions were adjusted as needed. Following the initial coding process, inductive reasoning was used to extract results closely aligned with participant-identified priorities.

## 3. Results

The analyzed reports (*n* = 375) gather the opinions, feelings, demands, and proposals of the participants aimed at improving their quality of work life and as a means to reduce the distress generated by working on the front line during the pandemic, with the constant risk of contagion and the imposed social isolation. The results have been compiled into two tables. Table 2 presents the set of emotions and thoughts alongside the proposed solutions and coping strategies, while Table 3 outlines the set of suggested demands for improving their quality of work life.

As observed in Table 2, the set of emotions and thoughts can be grouped into 8 major categories ranging from fear of personal, family, and patient contagion; to the confinement situation during shifts leading to social distancing and absence of outdoor leisure; sensation of lack of control and insecurity, causing anxiety symptoms; feeling of unclear procedures and inadequate protective equipment, resulting in frustration towards management; distrust towards colleagues and other services; stress during shifts; social pressure; and profession-related dilemmas. Additionally, the study highlights the solutions and coping strategies proposed by emergency medical professionals to address these needs. These solutions include specific measures to enhance safety during services, promote social contact and leisure at home, manage stress and anxiety, establish clear procedures and effective communication, as well as improve empathy towards patients and their families.

## 4. Discussion

The results indicate that the objectives were achieved. The opinions, feelings, demands, and proposals of coping strategies of the participants were collected with the aim of improving their quality of work life and reducing the distress generated by working on the front line during the pandemic. The coping measures and strategies proposed by prehospital healthcare professionals, as well as the demands suggested to improve their quality of work life, reflect a deep understanding of the challenges posed by the pandemic. The proposed solutions and coping techniques directly address the emotions and concerns expressed by the participants, such as fear of contagion, lack of clarity in procedures, frustration towards management, distrust towards colleagues and other services, shift-related stress, and professional dilemmas.

The emotions delineated in Table 2 align closely with Scott et al. [1], who indicated that recurrent exposure to stressful and potentially traumatic incidents significantly impacts the physiological and psychological well-being of healthcare practitioners. Within this investigation, healthcare personnel articulated apprehensions concerning contagion, both personally and among their families. This apprehension underscores the psychological strain stemming from the continual jeopardy of exposure to infectious pathogens, thereby substantiating the hypothesis that iterative exposure to such stressors exerts a profound toll on mental resilience. Moreover, frustrations regarding the absence of clear procedural directives and insufficient personal protective equipment serve to affirm this. These frustrations signify a pervasive sense of vulnerability and insecurity among healthcare professionals, arising from perceived deficiencies in resource allocation and support infrastructure. Such sentiments of inadequacy may exacerbate the emotional burden of their vocation, thereby reinforcing the conjecture concerning the detrimental repercussions of recurrent exposure to distressing circumstances.

**Table 2.** Identified emotions, thoughts, proposed solutions, and coping strategies.

**Categories**	**Emotions & thoughts**	**Solutions and coping strategies**
Fear of contagion and of infecting others	Fear of own contagion. Fear of family contagion, especially in children and older people. Feelings of guilt for exposing their family due to their profession. Feeling that family members do not understand the seriousness of the situation, leading to non-compliance with confinement or exposure without adequate protection. Fear and sense of responsibility for continuing to transport vulnerable patients (dialysis, chemotherapy, etc.).	***Increase safety during services:*** Assist each other in wearing PPE. Use the intercom to request information from family members and explain the procedure to be followed. Maintain distance from patients and offer them masks and gloves to wear. Cleaning and disinfection of the ambulance after each service. ***Increased safety at the base***: Create a clean area. Bleach-infused mat to clean boots. Constant hand hygiene. Wear a mask throughout the shift, also at the base. Divide tasks to keep the base clean at all times. If possible, sleep-in separate rooms at the base. Eat one by one or maintain a safe distance. ***Increased safety outside of work***: Shower at work and go home with different clothes. Cleaning and disinfection of the home. Use safety measures when going shopping. Raise awareness among friends and family to protect themselves.
Confinement situation among guards leading to social distancing and lack of outdoor leisure.	Leisure among guards. Distance from friends and family, causing sadness.	Facilitate social and family contact through technology. Promote new indoor leisure activities or adapt existing ones. Increase calls among colleagues. Plan activities in advance to facilitate participation. Focus on healthy eating as a way to stay present: make shopping lists, go shopping, cook, etc. Encourage communication with cohabitants and take actions to stay active: board games, puzzles, etc. Set aside time to be alone and listen to oneself; if necessary, introduce meditation techniques or other anxiety control techniques. Encourage physical activity at home. Find new routes home as a way to stay focused on the present.
Feeling of lack of control and insecurity, leading to anxiety symptoms	Feeling of lack of control over the situation, doubt about one's own professionalism. Engaging in irrational protective behaviors. Lack of motivation to work due to a sense of uncertainty. Anticipation of overwhelmed care: Anxiety about sick leave and anticipation of extending shifts to cover absences. Need for excessive information. Anticipatory anxiety: Trouble sleeping before shifts. Increased irritability with family and colleagues.	Remember the reasons and purpose of work before starting. Maintain a positive language, focused on practical solutions for what we control. Avoid anticipation, focus on the present. Avoid excessive information: Limit sources of information. Prepare interventions with colleagues in advance. Share specific experiences with colleagues to imagine actions and anticipate them. More task structuring among colleagues, plan responsibilities for each one. Perform actions more slowly to ensure safety.
Feeling of lack of clear procedures and inadequate PPE leading to frustration towards management	Uncertainty about changes in procedures. Feeling of powerlessness, anger, and injustice due to feeling under-equipped to work properly. Lack of PPEs. Frustration about justifying the materials they use. Perception of being put at risk and feeling poorly protected by superiors. They believe they need more serological tests to ensure they are not infected. Unfamiliarity with some new protocols (e.g., disinfection of work clothing). They mention that, due to lack of resources, basic units are used where more advanced units would be required.	***Clear procedures:*** Read and summarize the most updated protocols and pass them on to other colleagues. Encourage increased communication with superiors. Find a spokesperson who has open communication with management and speaks for everyone. Call a meeting for doubts or discrepancies with other healthcare units. Find spaces to explain the new procedures to colleagues who are on vacation or on leave. Nurses and doctors are assumed to be the first point of reference for doubts in procedures and the correct use of PPEs. Have visual instructions in important areas for quick check-ups. ***Promote responsible self-management:*** Use PPEs that correspond to each service, neither too much nor too little. Create and recycle PPEs: Create fabric masks to wear over the hygienic one and thus recycle. Creation of protective screens with 3D printers. Volunteers create gowns from garbage bags.
Mistrust towards other colleagues and services	Feeling that some colleagues are not afraid of contagion and do not protect themselves. Feeling that they do not perform disinfection correctly. Some report thefts of protective equipment. Complaints that primary care staff are not doing their job. Complaints that hospital staff do not provide them with the information they need to perform transfers.	Trust in the experience and professionalism of colleagues. Congratulate, thank, and motivate each other. Avoid complaint and confrontation. More communication between units and bases. Be open to listening, sharing, expressing opinions, and accepting proposals from others even if they are not experts in the field. Facilitate the adaptation of professionals coming from other areas: offer open communication and explain what they require. Clean the ambulance and the base at the beginning of work.
Stress during the shift	Pressure regarding making mistakes that could become a source of contagion. Sustained stress during the workday. Fatigue from new hygiene measures and discomfort from wearing a mask. Considerable increase in workload, with many activations and transfers daily. Anticipation of what is to come. Constant state of alertness.	***More communication and support among colleagues***: Expressing discomfort and concerns openly. More rest time between shifts to communicate. More time between shift changes to communicate. Use of humor as a strategy for managing stress and anxiety. Dealing with disruptive or anxious colleagues, preventing the spread of fears, frustration, or concerns to other colleagues. ***Knowing when to stop:*** In situations of emotional overwhelm, use anxiety control techniques such as relaxation, breathing, etc. Emotional disengagement at the end of shifts, establishing transition periods. If necessary, turn off the phone for a few hours on days off.
Social pressure	Social pressure as a collective. Feeling constantly demanded by friends and family due to their profession. Fear of being blamed by neighbors in small towns.	Transmit messages of calm to the population. Provide specific guidelines to friends and family. Use appropriate protective measures to set an example.
Professional Dilemmas	Feeling of strangeness and dehumanization towards patients due to lack of contact. The need for protection takes away valuable early attention time (e.g., during a PCR). Disturbance regarding the limitation of therapeutic effort. Sometimes only one of the two technicians interacts to reduce exposure, the other feels bad for not being able to help. Some patients call the ambulance when they are very ill, out of fear of going to the hospital.	Foster a warmer and more humanizing contact with patients (increased communication). Provide more explanations to family members. Emphasize the importance of saying goodbye to patients. Remind patients to take their mobile phone and charger to communicate with family members. Request a mobile phone number from family members in case they need to make communications from the hospital. When only one technician interacts to reduce exposure, the other remains in a place where they can have auditory or visual contact with the companion, to assist if required.

**Table 3.** Demands requested by the collective.

**Category of demand**	**Requests**
Need for familiarity	Maintain regular colleagues. Work in the usual base.
Need for information and attention	Clear and sincere explanation from superiors. Clear reference points to ask questions. Channel for complaints. To be heard by superiors, with greater attention and quality. Psychological support.
Need for education	Learn techniques to manage emotions of patients and family members. More tools for communication with patients and family members. More educational resources for complex situations. More information on proper disinfection.
Need for more testing	More serological tests for healthcare personnel.
Changes in the usual mode of operation	24-hour shifts are excessive due to workload and required attention. Cleaning and disinfection of ambulances, work clothes, and spaces by trained professionals. Additional assistance from TES to support nursing. Surprise audits of subcontracted companies to ensure compliance with protocols and occupational safety.
Psychologist staff	Increased presence of psychologist staff. Additional tools to manage fear and distress.

The results obtained in this study are in line with those of other researchers [1]–[3] regarding the significance of clinical debriefing sessions in addressing the distress and stress experienced by healthcare professionals. Feelings of frustration, mistrust towards colleagues and services, stress during shifts, and professional dilemmas highlighted in Table 2 are consistent with the challenges discussed by Scott et al. [1], Nocera & Merritt [2], and Rose & Cheng [3], who emphasize the importance of fostering supportive work environments, promoting effective communication, and implementing strategies to address the psychological well-being of workers. On other hand, the strategies outlined, such as enhancing safety measures during services and implementing clear procedures, are indicative of coping mechanisms advocated by Nocera & Merritt [2] and Rose & Cheng [3] to mitigate the emotional toll of healthcare work. Therefore, the congruence between the findings of this study and the existing literature underscores the critical role of clinical debriefing sessions in addressing distress and stress among healthcare professionals, thereby emphasizing the importance of ongoing support mechanisms within healthcare settings.

The TALK model selected for this intervention has shown positive outcomes. This model stands out for its emphasis on needs analysis and the search for practical solutions, making it particularly suitable for addressing the challenges faced by healthcare professionals during critical events [1]. The decision to select the TALK clinical debriefing model was grounded in addressing the inconsistencies and limitations associated with existing debriefing approaches. While the Critical Incident Stress Debriefing (CISD) has been widely utilized [4], its effectiveness in preventing post-traumatic stress disorder (PTSD) and other psychological consequences has been questioned [7]–[9]. On the other hand, recent systematic reviews have underscored the effectiveness of customized interventions such as clinical debriefing in meeting the specific needs of recipients [10], but this approach is applied individually and may not be suitable for group interventions like the ones we are discussing here.

Additionally, unlike CISD, which primarily focuses on emotional processing, TALK sessions provide a structured framework for identifying and addressing practical concerns and enhancing team resilience [11]. By transitioning into operational meetings, TALK sessions enable participants to review decisions, identify learning points, and offer personal reflections, fostering a shared narrative of the event and providing peer support [1]. Thus, in this study, as depicted in Tables 2 and 3, the intervention comprehensively addressed the specific needs and demands of healthcare professionals. On one hand, it allowed healthcare staff to identify and address emotions, thoughts, and concerns in a structured manner and provide concrete coping solutions and strategies for each of these categories. This contributed to increasing the sense of control and security, thereby reducing the anxiety and stress experienced by professionals during the pandemic. Furthermore, as shown in Table 3, it exposed specific demands of the collective, such as the need for familiarity, information, psychological support, and education. Collectively, these findings support the conclusion that the TALK model is useful for addressing the needs and demands of the healthcare professional collective, thereby strengthening their ability to face challenges during periods of crisis.

The acceptance of this intervention also suggests that workers in the Catalan emergency medical system value the support provided by management following traumatic incidents such as the pandemic. This result is consistent with the findings of Richins et al. [10] in their 2020 scoping review of early post-traumatic interventions among emergency personnel. It is worth noting that participation in this study was voluntary, which may account for the positive results obtained. This significance was emphasized by Billings et al. [22] in their systematic review of debriefing interventions following traumatic incidents in the workplace, where they found that studies mandating debriefing tended to yield less favourable effectiveness and acceptability outcomes. Additionally, to achieve positive outcomes, such interventions necessitate the involvement of professionals trained in mental health [22],[23], as was the case in this study, where the interventions were conducted by psychologists affiliated with the emergency medical service.

The selection of the TALK clinical reporting model was grounded in its alignment with the needs of healthcare professionals, its structured approach to addressing practical concerns, and its potential to promote peer support and resilience. Psychologists' observations suggest that participants were able to effectively embrace the “TALK debrief” model and engage in open and constructive dialogue, indicating that the sessions successfully facilitated communication and addressed the practical needs of staff. This underscores the effectiveness of the ‘TALK debrief’ approach in providing emotional support and fostering open communication for processing traumatic experiences following critical events. The accessibility and user-friendliness of the TALK model, coupled with the positive outcomes observed, render it a pragmatic option for healthcare settings where time and resources may be constrained [5]. These findings are consistent with earlier observations, which highlight that its structured format ensures the inclusion of all personnel affected by critical events, thereby promoting interprofessional collaboration and facilitating a comprehensive understanding of the circumstances and impact of such events [2],[17].

### 4.1. Study limitations

This study has several limitations that should be considered. First, the experiences and needs of prehospital staff may vary between regions and countries; therefore, the findings may not be fully applicable to other populations or contexts. Second, there is the possibility of social desirability bias, and participants may have provided answers they believed were socially desirable. Third, there was no long-term follow-up, so the effectiveness or sustained impact on workplace well-being remains unknown. Finally, although a qualitative methodology was used to collect data, the subjective nature of the responses may make interpretation and comparison of the results challenging.

### 4.2. Future research

To delve deeper into the needs and solutions of prehospital staff during health crises and overcome the limitations of the current study, longitudinal research could be undertaken to understand how TALK debriefing affects their long-term work well-being. Additionally, conducting comparative studies across different countries or regions would be insightful to identify similarities and differences in addressing challenges during health crises. Augmenting qualitative findings with quantitative research would enable the measurement of the impact of psychological and supportive interventions on mental health, stress levels, and job satisfaction.

## 5. Conclusions

The results of this study indicate that the TALK clinical reporting model facilitated open and constructive communication among healthcare staff during debriefing sessions, allowing for the identification and application of practical strategies to address their concerns. This suggests that the sessions not only provided emotional support, but also promoted collaboration and the exchange of ideas between colleagues. In turn, formalizing the debriefing sessions provided a platform for healthcare staff to express and validate their concerns, allowing them to feel heard and supported by their institution and their colleagues. This underscores the importance of prioritizing self-care and staff support during crisis situations.

In conclusion, the study underscored the utility of the TALK clinical debriefing as an effective tool for promoting open and constructive communication among prehospital staff. This approach enabled professionals to share experiences, express emotions and thoughts, and collectively identify coping strategies and practical solutions. The findings emphasized the importance of addressing comprehensively the well-being and working conditions of these professionals during health crisis situations.

## Use of AI tools declaration

The authors declare they have not used Artificial Intelligence (AI) tools in the creation of this article.
